# The Assessment of Dietary Organic Zinc on Zinc Homeostasis, Antioxidant Capacity, Immune Response, Glycolysis and Intestinal Microbiota in White Shrimp (*Litopenaeus vannamei* Boone, 1931)

**DOI:** 10.3390/antiox11081492

**Published:** 2022-07-29

**Authors:** Jinzhu Yang, Tiantian Wang, Gang Lin, Mingzhu Li, Yanjiao Zhang, Kangsen Mai

**Affiliations:** 1 The Key Laboratory of Aquaculture Nutrition and Feed (Ministry of Agriculture), The Key Laboratory of Mariculture (Ministry of Education), Ocean University of China, Qingdao 266003, China; yangjinzhu@stu.ouc.edu.cn (J.Y.); 21180511037@stu.ouc.edu.cn (T.W.); kmai@ouc.edu.cn (K.M.); 2 Institute of Quality Standards and Testing Technology for Agricultural Products, Chinese Academy of Agricultural Sciences, Beijing 100081, China; lingang@caas.cn; 3 College of Agriculture, Ludong University, Yantai 264025, China; 2982@ldu.edu.cn

**Keywords:** organic zinc, zinc homeostasis, antioxidants, immunity, glycolysis, intestinal microbiota, *Litopenaeus vannamei* Boone, 1931

## Abstract

This study aimed to assess dietary organic zinc on zinc homeostasis, antioxidant capacity, immune response, glycolysis and intestinal microbiota in white shrimp (*Litopenaeus vannamei* Boone, 1931). Six experimental diets were formulated: Control, zinc free; S120, 120 mg·kg^−1^ zinc from ZnSO_4_·7H_2_O added into control diet; O30, O60, O90 and O120, 30, 60, 90 and 120 mg·kg^−1^ zinc from Zn-proteinate added into control diet, respectively. The results showed that organic zinc significantly promoted zinc content and gene expression of ZnT1, ZIP11 and MT in the hepatopancreas and enhanced antioxidant capacity and immunity (in terms of increased activities of T-SOD, Cu/Zn SOD, PO, LZM, decreased content of MDA, upregulated expressions of GST, G6PDH, ProPO, LZM and Hemo, and increased resistance to *Vibrio parahaemolyticus*). Organic zinc significantly upregulated GluT1 expression in the intestine, increased glucose content of plasma and GCK, PFK and PDH activities of hepatopancreas, and decreased pyruvate content of hepatopancreas. Organic zinc improved intestinal microbiota communities, increased the abundance of potentially beneficial bacteria and decreased the abundance of potential pathogens. Inorganic zinc (S120) also had positive effects, but organic zinc (as low as O60) could achieve better effects. Overall, organic zinc had a higher bioavailability and was a more beneficial zinc resource than inorganic zinc in shrimp feeds.

## 1. Introduction

As an essential microelement for animals, zinc is well known for its key role in various physiological processes such as organism growth and metabolism of proteins, carbohydrates and lipids [1,2]. Zinc is also a cofactor or component of various enzymes related to antioxidants, immune response and regulation, such as alkaline phosphatase (AKP), metallothionein (MT), superoxide dismutase (SOD) and copper/zinc superoxide dismutase (Cu/Zn SOD) [3]. In aquafeeds, zinc sulfate is traditionally used to meet the demand of aquatic animals due to its lower price and easy accessibility [4,5]. However, the disadvantages of zinc sulfate have been noticed with its low bioavailability and potential environmental problems. On the one hand, plant-based ingredients have been widely used in aquafeeds [6,7,8]. It is known that plant-based ingredients contain antinutritional factors that reduce the bioavailability of minerals [9,10]; for example, phytate is easily chelated to Zn^2+^, forming stable chelates that are not absorbed intestinally [3,9]. Thus, overdosage of zinc addition is common in aquafeeds [11,12], which increases the risk of water pollution in intensive aquaculture zones and their surroundings. On the other hand, sulfate salts are a common type of inorganic mineral (e.g., ZnSO_4_, CuSO_4_, MnSO_4_) in feed addition [13]. The high concentration of SO_4_^2−^ is toxic to aquatic organisms. SO_4_^2−^ can be reduced into sulfide under anaerobic conditions, which is toxic to a number of aquatic organisms as well [14,15].

Organic zinc, formed by the chelation of Zn^2+^ with amino acids (e.g., glycine, methionine) and/or partially hydrolyzed proteins, provides a new option for zinc supplementation in aquafeeds [16]. It has been reported that organic zinc is more easily absorbed by the intestinal epithelium than inorganic zinc [17,18], and it can prevent the chelation of Zn^2+^ with phytate and protect the micronutrients (e.g., vitamins, fatty acids) from oxidation by Zn^2+^ [19]. In channel catfish (*Ictalurus punctatus*), organic zinc had over 3 times the potency of inorganic zinc in improving growth and increased resistance to *Edwardsiella ictaluri* [20,21]. Similarly, there was also a higher bioavailability of organic zinc than inorganic zinc in beluga sturgeon (*Huso huso*) [22], pangasius catfish (*Pangasianodon hypophthalmus*) [23] and juvenile abalone (*Haliotis discus hannai* Ino.) [24]. In triploid rainbow trout (*Oncorhynchus mykiss*), organic zinc reduced the zinc requirement and enhanced antioxidant capacity compared to inorganic zinc [25,26]. In white shrimp (*Litopenaeus vannamei* Boone, 1931), compared to inorganic zinc, organic zinc promoted growth performance and enhanced immunity and resistance to *Vibrio harveyi* better [27,28,29]. Glycolysis is a cellular process that breaks down glucose into pyruvate. Pyruvate can enter the tricarboxylic acid cycle (TAC) for further ATP production. Glycolysis is also an important method to metabolize glucose in most organisms. Previous studies have described that zinc plays a role in stimulating glycolysis in mice [30] and rats [31,32]; however, in aquaculture, relevant information is limited. The intestinal microbiota plays key roles in host health, nutrition metabolism and immune response. Studies have found zinc could increase the abundance of potential probiotics in weanling pigs [33] and improve the microbial population of broiler chickens [34]. However, information on the effect of zinc on the intestinal microbiota of aquatic animals is also limited.

White shrimp is a major species in aquaculture around the world and has great economic value due to its great texture, rapid growth rate and good adaptability to the environment [35]. At present, the increasing farming density and scale pose a challenge to the health and disease resistance of shrimp [36]. Organic zinc is considered to have immune-enhancing effects, which is beneficial for shrimp health. Most previous studies of organic zinc have focused on growth performance and biochemical parameters. This study aimed to assess dietary organic zinc (Zn-proteinate) on zinc homeostasis, antioxidant capacity, immune response, glycolysis, intestinal microbiota and resistance to *V. parahaemolyticus* in white shrimp, which will provide a comprehensive assessment of organic zinc application in shrimp culture.

## 2. Materials and Methods

### 2.1. Experimental Diets

Six isonitrogenous and isolipidic experimental diets were formulated to contain different dosage forms of zinc, resulting in the following six dietary treatments: Control, dietary Zn free; S120, dietary 120 mg·kg^−1^ zinc from ZnSO_4_·7H_2_O added into control diet; O30, O60, O90, O120, dietary 30, 60, 90, 120 mg·kg^−1^ zinc from Zn-proteinate added into control diet, respectively. All the ingredients were thoroughly mixed and pelleted with an approximate diameter of 2 mm. After that, feeds were dried until constant weight at 55 °C in a ventilated oven. Feeds were then stored at −20 °C until use. The formulation, chemical composition and zinc content in feeds are shown in Table 1.

Analysis of the chemical compositions of feeds was performed following standard protocols [37]. Dry matter was measured by drying samples to a constant weight at 105 °C, crude protein was determined by measuring nitrogen (N × 6.25) using the Kjeldahl method (FOSS 8400, Denmark, Sweden), crude lipid was determined by mineral ether extraction using the Soxhlet method (BUCHI 36880, Flawil, Switzerland) and ash content was determined by incineration of samples at 550 °C in a muffle furnace.

The moisture was calculated with the following equation:

Moisture (%) = 100 × (W_1_ − W_2_)/W_1_, (1)

W_1_: Wet weight of matter; W_2_: Dry weight of matter.

The ash content was calculated with the following equation:

Ash (%) = 100 × W_3_/W_4_, (2)

W_3_: Ash weight after incineration; W_4_: Dry weight before incineration.

### 2.2. Feeding Trial and Sample Collection

The feeding trial was carried out at Huanghai Aquaculture Co., Ltd., Shandong, China. White shrimp were purchased from a local farm. Before the feeding trial, shrimp were acclimated to a commercial diet for 2 weeks with flowing water. Shrimp were then fasted for 24 h and weighted. A total of 960 shrimp (initial body weight of 2.37 ± 0.01 g) were randomly distributed to 24 cylindrical fiberglass tanks (with 200 L of seawater) in an indoor rearing system with flow-through seawater. A total of 40 shrimp were cultured in each tank. The six diets were randomly assigned to tanks (4 replications each group). During the feeding trial, shrimp were fed 4 times daily (05:30, 11:00, 16:30 and 21:30). The daily feeding quantity was at 4–6% of body weight and adjusted according to previous feeding responses. Two-thirds of seawater was exchanged twice daily. The water condition was detected once a week, and the results were as follows: temperature was 26.4–28.0 °C; salinity was 31–33‰; pH was 8.2–8.4; dissolved oxygen was higher than 7 mg·L^−1^; nitrite was lower than 0.005 mg·L^−1^; nitrate was lower than 15 mg·L^−1^; ammonia was lower than 0.02 mg·L^−1^.

After 8 weeks of feeding, shrimp were fasted for 24 h; all surviving shrimp were collected, and then the body length and weight of each shrimp were measured. After that, 12 shrimp of similar size from each tank were randomly selected. The hemolymph was collected from the caudal vein using 1 mL syringes and diluted immediately at a hemolymph-to-anticoagulant ratio of 1:1.5 (10 mmol·L^−1^ EDTA-Na_2_, 450 mmol·L^−1^ NaCl, 10 mmol·L^−1^ KCl, 10 mmol·L^−1^ HEPES, pH 7.3) [38]. Approximately 50 μL of hemolymph was used for the hemocyte count using a hemocytometer (XB-K-25, Yuhuan County Qiujing Medical Instrument Factory, Zhejiang, China). The rest was centrifuged at 4 °C, 500 g·min^−1^ for 10 min. The supernatant was then collected into 0.2 mL PCR tubes (Cat. No.: PCR02C, Axygen™, Union City, CA, USA) and stored at −80 °C for biochemical analysis. After that, the hepatopancreas was quickly removed and then transferred to 1.8 mL sterile RNase-free cryogenic tubes (Cat. No.: 377267, Nunc™, Rochester, NY, USA), frozen in liquid nitrogen and stored at −80 °C for analysis of enzyme activity and gene expression. Intestines were then removed and transferred to 1.8 mL sterile RNase-free cryogenic tubes (Cat. No.: 377267, Nunc™, Rochester, NY, USA), frozen in liquid nitrogen and stored at −80 °C, of which 6 were used for gene expression analysis and the other 6 for microbiota analysis. The muscle and carapace of 2 shrimp were randomly selected and stored at −20 °C for zinc concentration analysis.

### 2.3. Vibrio Parahaemolyticus Challenge

After the feeding trial, 30 shrimp of each group were randomly selected and separated into 3 tanks (with 50 L of seawater). Each shrimp was injected intramuscularly with 0.1 mL of *Vibrio parahaemolyticus* (5 × 10^6^ CFU/mL). Based on pre-experiments, mortality was recorded for 7 days. During the challenging trial, temperature was 26.4–28.0 °C, salinity was 31–33‰, pH was 8.2–8.4, dissolved oxygen was higher than 7 mg·L^−1^, nitrite was lower than 0.005 mg·L^−1^, nitrate was lower than 15 mg·L^−1^ and ammonia was lower than 0.02 mg·L^−1^.

### 2.4. Growth Performance and Hemocyte Count

Growth performance was calculated by using the following equation:

Weight gain rate (WGR,%) = 100 × (final body weight − initial body weight)/initial body weight(3)

Specific growth rate (SGR,%·day^−1^) = 100 × (Ln final body weight − Ln initial body weight)/days (4)

Feed intake (FI,%·day^−1^) = 100 × feed intake/[(initial body weight + final body weight)/2]/days (5)

Feed efficiency (FE) = (final body weight − initial body weight)/feeds consumed(6)

Condition factor (CF, 100 g·cm^−3^) = 100 × final body weight/finial body length^3^ (7)

Hemocyte count was measured with a hemocytometer under a light microscope (Nikon, E 600, Tokyo, Japan). The number of blood cells per mL of hemolymph was calculated according to the formula below:

Hemocyte count in 1 mL hemolymph = *A*/5 × 25 × 10000 × *B* (8)

*A*: the total hemocyte count in 5 medium squares; *B*: dilution ratio of the sample.

### 2.5. Zinc Accumulation Analysis

Zinc concentrations in the diets, muscle and carapace were analyzed by an inductively coupled plasma optical emission spectrometer (ICP-OES, PE 2100DV, Perkin Elmer, Boston, MA, USA). This analysis was completed by the Beijing ZKGX Research Institute of Chemical Technology (Material Lab, Beijing, China). Zinc concentrations in the plasma and hepatopancreas were determined with a commercial assay kit (E011-1-1; Nanjing Jiancheng Bioengineering Institute, Nanjing, China).

### 2.6. Biochemical Analysis

Plasma of shrimp was used for biochemical analysis directly. The hepatopancreas was weighted, thawed and homogenized (1:9) in ice-cold 0.9% NaCl solution. After centrifugation (2500 rpm, 15 min, 4 °C), the supernatant was collected for biochemical analysis. The activities of acid phosphatase (ACP, A060-2-1), AKP (A059-2-2), catalase (CAT, A007-1-1), phenoloxidase (PO, H247), total antioxidant capacity (T-AOC, A015-2-1) and the contents of glucose (Glu, A154-1-1), malondialdehyde (MDA, A003-1-2) of plasma were determined with commercial assay kits (Nanjing Jiancheng Bioengineering Institute, Nanjing, China). The content of pyruvate (A081-1-1) and the activities of glucokinase (GCK, H439-1), phosphofructokinase (PFK, H244), pyruvate dehydrogenase (PDH, H262-1-2) of hepatopancreas were determined with commercial assay kits (Nanjing Jiancheng Bioengineering Institute, Nanjing, China). The activities of total superoxide dismutase (T-SOD, S0101M) and Cu/Zn SOD (S0103) of plasma and hepatopancreas and the content of total protein (P0012) of hepatopancreas were determined with commercial assay kits (Beyotime Biotechnology, Shanghai, China). The activity of lysozyme (LZM) of plasma was determined by using commercial Shrimp ELISA kits and following the manufacturer’s instructions (CK-E94755, Shanghai Elisa Biotech Co., Ltd., Shanghai, China).

### 2.7. RNA Extraction and qPCR

The total RNA of hepatopancreas and intestine were extracted using the MolPure^®^ Cell/Tissue Total RNA Kit (19221ES50; Yeasen Biotechnology (Shanghai) Co., Ltd., Shanghai, China). The concentration and quality of RNA were assessed with NanoDrop™ 2000 spectrophotometers (Thermo Scientific™, Waltham, MA, USA). The integrity of extracted RNA was determined by electrophoresis on a 1.2% (*w*/*v*) agarose gel. Reverse transcription of 1000 ng of RNA was conducted using Hifair^®^ III 1st Strand cDNA Synthesis SuperMix for qPCR (11141ES60; Yeasen, Shanghai, China).

The qPCR was performed in a 20 μL volume: 1 μL of cDNA template (≤50 ng); 0.4 μL of forward primer (10 μM); 0.4 μL of reverse primer (10 μM); 8.2 μL of RNase-free ddH_2_O (P071-01, Vazyme Biotech Co., Ltd., Nanjing, China); 10 μL of SYBR^®^ Green Premix Pro Taq HS qPCR Kit (AG11701, Accurate Biotechnology (Hunan) Co., Ltd., Hunan, China). A two-step qPCR program was used: 95 °C for 30 s, followed by 40 cycles of 95 °C for 5 s and 60 °C for 30 s. Finally, melting curve analysis was used to ensure the specification of the PCR product. Specific gene primers were designed in NCBI, synthesized by Sangon Biotech (Shanghai) Co., Ltd., Shanghai, China. The specificity and amplification efficiency of primers were assessed (Table S1). β-Actin, 18S ribosomal RNA and elongation factor 1-alpha were selected as candidate housekeeping genes. BestKeeper [39] and NormFinder [40] tools were used to assess the most stable expression gene. Finally, β-actin was assessed as the best housekeeping gene in the present experiment (Tables S2 and S3).

All qPCR analyses were performed in CFX96 Touch Real-Time PCR Detection System (Bio-Rad, Hercules, CA, USA). Moreover, a no-template control (NTC) (0 μL of cDNA template, 0.4 μL of forward primer (10 μM); 0.4 μL of reverse primer (10 μM); 9.2 μL of RNase-free ddH_2_O; 10 μL of SYBR^®^ Green Premix Pro Taq HS qPCR Kit) was set in each qPCR plate reaction to ensure no extraneous nucleic acid contamination. The gene expression levels were normalized using the relative quantitative method (2^−ΔΔCq^) referencing β-actin of shrimp [41].

### 2.8. Intestinal Microbiota DNA Extraction and Sequencing

Genomic DNA of intestinal microbiota was extracted using the QIAamp PowerFecal^®^ Pro DNA Kit (51804, Qiagen, Hilden, Germany) on a super-clean bench following the manual. Primer 515F/806R was used to amplify the V4 region of the 16S rRNA gene. PCR reaction and quality control were performed by Novogene Genomics Technology Co., Ltd., Beijing, China. Sequencing was conducted on an Illumina NovaSeq platform provided by Novogene Genomics Technology Co., Ltd., Beijing, China. After sequencing, raw data were merged with Fast Length Adjustment of SHort reads (FLASH) [42] and then assigned to each sample with unique barcodes. To obtain effective reads for further analysis, Cutadapter was used to cut adapter, barcode and primer sequences and filter low-quality reads [43], and the UCHIME algorithm was used to detect and remove chimeric sequences [44]. After dereplication, abundance sort and discarding singleton reads, sequences were clustered to operational taxonomic units (OTUs) with ≥97% similarity using UPARSE [45]. The representative sequence for each OUT was screened for further annotation using the Silva Database (version 138) based on the Ribosomal Database Project (RDP) classifier [46]. Alpha diversity (OTUs, Chao1, ACE, Shannon, Simpson and PD whole tree) and beta diversity (principal coordinates analysis (PCoA) and unweighted pair group method with arithmetic mean (UPGMA) clustering) were calculated with Quantitative Insights Into Microbial Ecology (QIIME) and displayed with R software (version 4.1.0) [47].

### 2.9. Statistical Analysis

Statistical software SPSS 22.0 for Windows (IBM SPSS Corporation, Chicago, IL, USA) was used for the data analysis. Results were analyzed by one-way analysis of variance (ANOVA). Tukey’s multiple-range test was used for the multiple comparisons of group means. Differences were regarded as significant when *p <* 0.05.

Microbiota sequence data analysis proceeded on the NovoMagic cloud platform provided by Novogene Genomics Technology Co., Ltd., Beijing, China. An analysis of molecular variance (AMOVA) test was employed to assess the difference of microbiota composition within or between groups using the adegenet package in R software (version 4.1.0). MetaStat analysis was used to identify the differential abundant taxa between groups. The *p*-value was adjusted by the Benjamini-Hochberg false discovery rate (FDR), and differences were regarded as significant when *Q* < 0.05 (*Q*, adjusted *p*-value) [48].

## 3. Results

### 3.1. Growth Performance

As shown in Table 2, the FBW, WGR and SGR of shrimp fed the O60 diet were significantly higher than those of shrimp fed the control diet (*p <* 0.05). S120, O30, O90 and O120 were intermediate in the FBW, WGR and SGR, with no significant differences observed (*p >* 0.05). No significant differences were observed in the FI, FE, CF and total hemocyte number of shrimp among all groups (*p >* 0.05).

### 3.2. Zinc Accumulation and Zinc Transport

**Figure 1:**
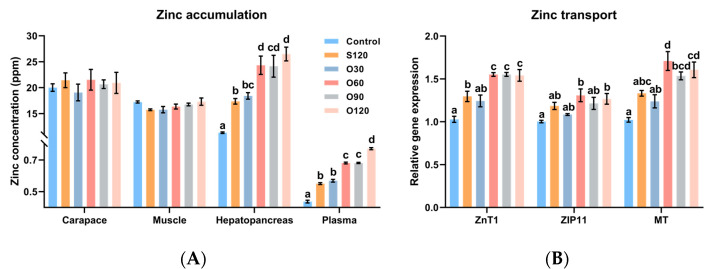
Effects of organic and inorganic zinc on zinc accumulation of *Litopenaeus vannamei* Boone, 1931 tissues (**A**) and gene expressions of zinc transport in hepatopancreas of *L. vannamei* (**B**). ZnT1, zinc transporter 1; ZIP11, zinc transporter ZIP11, MT, metallothionein. Values represented are means ± S.E. of 4 replicate tanks. ^a,b,c,d^ Value bars not sharing the same superscript letter are significantly different (*p <* 0.05).

As shown in Figure 1A, no significant differences were observed in the zinc concentrations of shrimp muscle and carapace among all groups (*p >* 0.05). The zinc concentrations in the hepatopancreas and plasma of the control group were lower than in all other diets (*p <* 0.05). The zinc concentrations in the hepatopancreas and plasma of shrimp fed the S120 diet were significantly lower than those of shrimp fed the O60, O90 and O120 diets (*p <* 0.05). The zinc concentration in plasma was the highest in the O120 group (*p <* 0.05).

The zinc-transport-related gene expressions in the hepatopancreas (Figure 1B) showed that, compared with the control diet, the expression of ZnT1, ZIP11 and MT was significantly upregulated by the S120, O60, O90 and O120 diets, the O60 and O120 diets, and the O60, O90 and O120 diets, respectively (*p <* 0.05). Compared with the S120 diet, the expression of ZnT1 and MT was significantly upregulated by the O60, O90 and O120 diets and the O60 diet, respectively (*p <* 0.05).

### 3.3. Antioxidant Capacity

**Figure 2:**
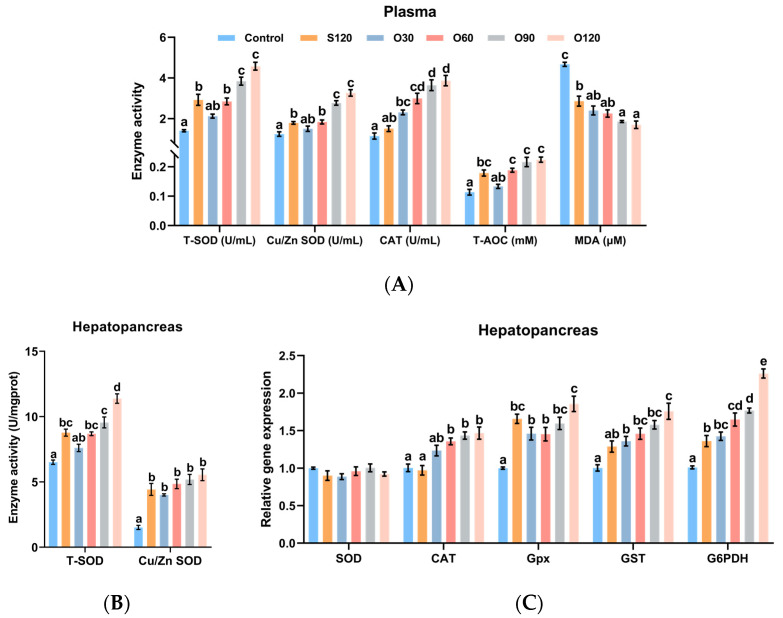
Effects of organic and inorganic zinc on antioxidant capacity of *L. vannamei*. (**A**) enzyme activities of plasma; (**B**) enzyme activities of hepatopancreas; (**C**) gene expressions of hepatopancreas. SOD, super dismutase; CAT, catalase; T-AOC, total antioxidant capacity; MDA, malondialdehyde; Gpx, glutathione peroxidase; GST, glutathione S-transferase; G6PDH, glucose-6-phosphate dehydrogenase. Values represented are means ± S.E. of 4 replicate tanks. ^a,b,c,d,e^ Value bars not sharing the same superscript letter are significantly different (*p <* 0.05).

In plasma, as shown in Figure 2A, the activities of T-SOD, Cu/Zn SOD, CAT and T-AOC of shrimp fed the O60, O90 and O120 diets were significantly higher than those of shrimp fed the control diet (*p <* 0.05). The activities of T-SOD, Cu/Zn SOD and CAT of shrimp fed the O90 and O120 diets were significantly higher than those of shrimp fed the S120 diet (*p <* 0.05). The activities of T-SOD, Cu/Zn SOD and T-AOC of shrimp fed the control diet were significantly lower than those of shrimp fed the S120 diet (*p <* 0.05). The MDA content of the control group was the highest among all groups (*p <* 0.05). The MDA contents of the O90 and O120 groups were significantly lower than those of the S120 group (*p <* 0.05). In the hepatopancreas, as shown in Figure 2B, the activities of T-SOD and Cu/Zn SOD of shrimp fed the S120, O60, O90 and O120 diets were significantly higher than those of shrimp fed the control diet (*p <* 0.05). The activity of T-SOD of the O120 group was the highest among all groups (*p <* 0.05).

The antioxidant-related gene expressions (Figure 2C) showed that the expressions of CAT, Gpx, GST and G6PDH of shrimp fed the O60, O90 and O120 diets were significantly higher than those of shrimp fed the control diet (*p <* 0.05). The expressions of Gpx and G6PDH of shrimp fed the S120 and O30 diets were significantly higher than those of shrimp fed the control diet (*p <* 0.05). When compared with the S120 diet, the expression of CAT was significantly upregulated by the O60, O90 and O120 diets, the expression of GST was significantly upregulated by the O120 diet, and the expression of G6PDH was significantly upregulated by the O60, O90 and O120 diets (*p <* 0.05). The expression of G6PDH of the O120 group was the highest among all groups (*p <* 0.05). No significant difference was observed in the expression of SOD among all groups (*p >* 0.05).

### 3.4. Immunity

**Figure 3:**
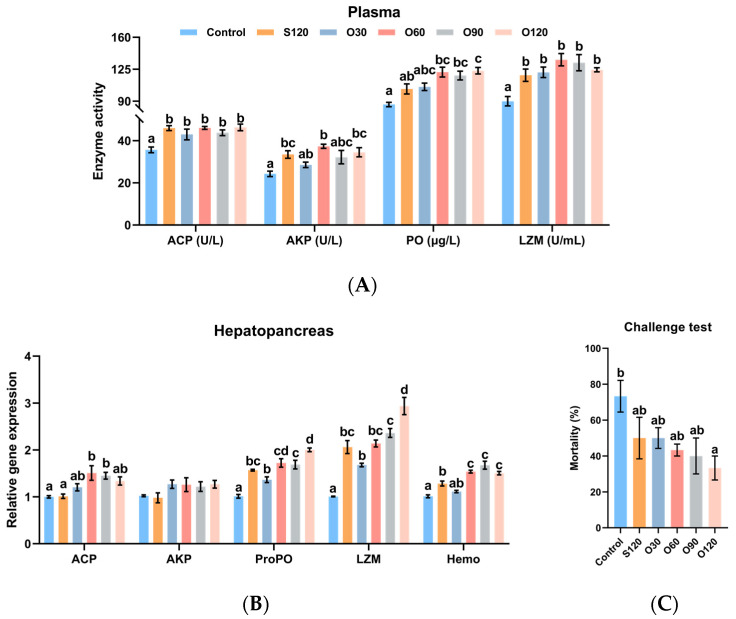
Effects of organic and inorganic zinc on immunity of *L. vannamei*. (**A**) enzyme activities of plasma; (**B**) gene expressions of hepatopancreas. (**C**) *Vibrio parahaemolyticus* challenge test of shrimp. ACP, acid phosphatase; AKP, alkaline phosphatase; PO, phenoloxidase; LZM: lysozyme; ProPO, pro-phenoloxidase; Hemo, hemocyanin. Values represented by A and B are means ± S.E. of 4 replicate tanks. Values represented by C are means ± S.E. of 3 replicate tanks. ^a,b,c,d^ Value bars not sharing the same superscript letter are significantly different (*p <* 0.05).

In plasma, as shown in Figure 3A, the activities of ACP and LZM in the control group was the lowest among all groups (*p <* 0.05) and no significant difference was observed among S120, O30, O60 and O120 groups (*p >* 0.05). The activities of AKP and PO of shrimp fed the O60 and O120 diets were significantly higher than those of shrimp fed the control diet (*p <* 0.05). The activity of AKP of shrimp fed the S120 diet was significantly higher than those of shrimp fed the control diet (*p <* 0.05). The activity of PO of shrimp fed the O120 diet was significantly higher than those of shrimp fed the S120 diet (*p <* 0.05).

The immunity-related gene expressions of hepatopancreas (Figure 3B) showed that the expression of ACP of shrimp fed the O60 and O90 diets was significantly higher than those of shrimp fed the control and S120 diets (*p <* 0.05). The expressions of ProPO, LZM and Hemo of shrimp fed the S120, O60, O90 and O120 diets were significantly higher than those of shrimp fed the control diet (*p <* 0.05). When compared with the S120 diet, the expression of ProPO and was significantly upregulated by the O120 diet, the expression of LZM was significantly upregulated by the O120 diet, and the expression of Hemo was significantly upregulated by the O60, O90 and O120 diets (*p <* 0.05). No significant difference was observed in the expression of AKP among all groups (*p >* 0.05).

The challenge test showed lower mortality of shrimp fed with supplementary dietary zinc after 7-day stress of *V. parahaemolyticus*. The mortality of shrimp fed the S120, O30, O60, O90 and O120 diets was decreased by 31.82%, 31.82%, 40.91%, 45.45% and 54.55% compared to the control, respectively, and the mortality in the O120 group was significantly lower than that of the control group (Figure 3C) (*p <* 0.05).

### 3.5. Glucose Transport and Glycolysis

**Figure 4:**
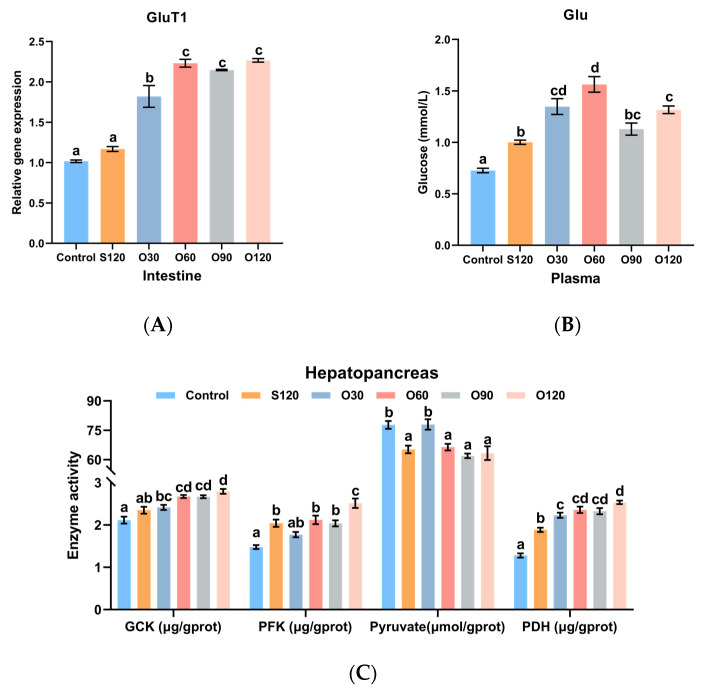
Effects of organic and inorganic zinc on transport and glycolysis of *L. vannamei*. (**A**) GluT1 expression of intestine; (**B**) Glu content of plasma; (**C**) enzyme activities of hepatopancreas. Glu, glucose; GluT1, glucose transporter 1; GCK, glucokinase; PFK, phosphofructokinase; PDH, pyruvate dehydrogenase. Values represented are means ± S.E. of 4 replicate tanks. ^a,b,c,d^ Value bars not sharing the same superscript letter are significantly different (*p <* 0.05).

As shown in Figure 4A, the expression of GluT1 in the intestine of shrimp fed the O30, O60, O90 and O120 diets was significantly higher than that of shrimp fed the control and S120 diets (*p <* 0.05). As shown in Figure 4B, the content of Glu in plasma of the control group was the lowest (*p <* 0.05). The content of Glu in plasma of the S120 group was significantly lower than the O30, O60 and O120 groups (*p <* 0.05). As shown in Figure 4C, the activities of GCK, PFK and PDH of shrimp fed the O60, O90 and O120 diets were significantly higher than those of shrimp fed the control diet (*p <* 0.05). When compared with the S120 diet, the activities of GCK, PFK and PDH of shrimp fed the O120 diet were significantly increased by the O60, O90 and O120 diets, the O120 diet, and the O30, O60, O90 and O120 diets, respectively (*p <* 0.05). The contents of pyruvate in the control and S120 groups were significantly higher than the other groups (*p <* 0.05).

### 3.6. Intestinal Microbiota

**Figure 5:**
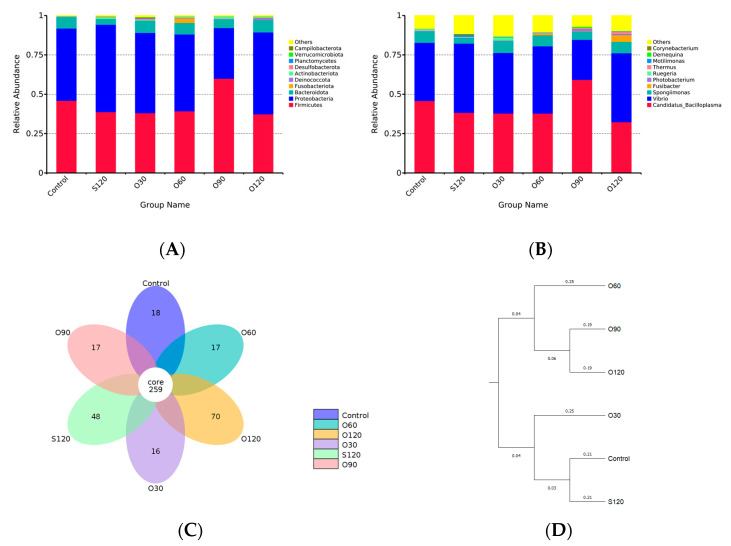

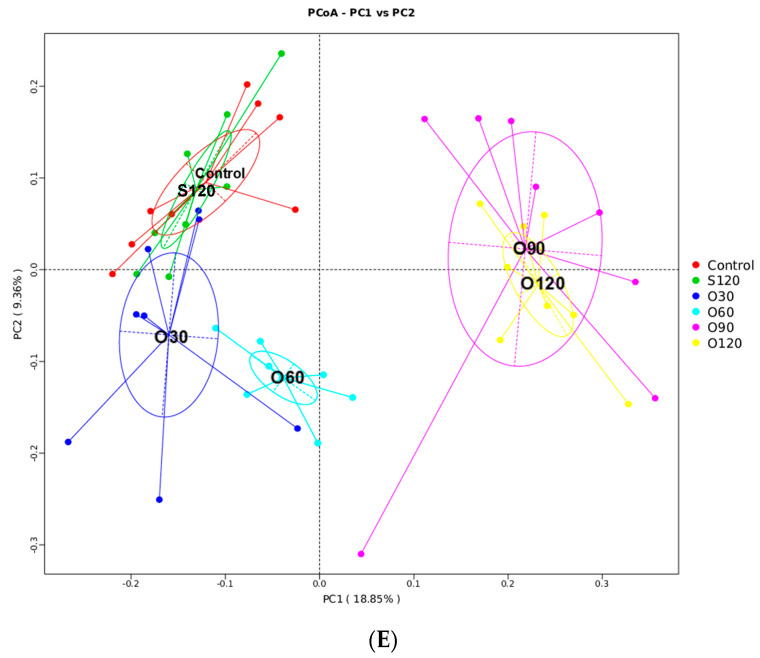
Effects of organic and inorganic zinc on intestinal microbiota of *L. vannamei*. Taxonomy classification of reads at phylum (**A**) and genus (**B**) levels. Only top 10 most abundant (based on relative abundance) bacterial phyla and genera were shown in the figures, other phyla and genera were all assigned as ‘Others’. Flower diagram of intestinal microbiota among all groups (**C**). UPGMA clustering trees in groups (**D**) and principal coordinate analysis (PCoA) plot in samples (**E**) based on unweighted UniFrac distances among all groups.

A total of 3,105,865 effective reads were obtained, and after annotation, 24 phyla, 42 classes, 109 orders, 204 families, 402 genera and 1454 OTUs were identified. Rank abundance, rarefaction curves and species accumulation boxplot showed that all samples reached the saturation phase, indicating adequate sequencing depth (Figure S1). At the phylum level, Firmicutes, Proteobacteria and Bacteroidota were the predominant bacterial phyla in the intestine among all groups (Figure 5A). At the genus level, *Candidatus_Bacilloplasma*, *Vibrio* and *Spongiimonas* were the predominant bacterial genera in the intestine among all groups (Figure 5B). As alpha diversity indices (OTUs, Chao1, ACE, Shannon, Simpson, PD whole tree) shown in Table 3, Chao1 and ACE indices in the O30 group were significantly higher than that in the O90 group (*p <* 0.05). Simpson index in the O120 group was significantly higher than that in the control and O90 groups (*p <* 0.05). No significant difference was observed in OTUs, Shannon index and PD whole tree among all groups (*p >* 0.05).

The flower diagram showed that all groups shared 259 OTUs, and the control, S120, O30, O60, O90 and O120 groups had 18, 48, 16, 17, 17 and 70 unique OTUs, respectively (Figure 5C). The AMOVA test confirmed the difference between groups was greater than the differences within groups (Table S4). UPGMA (Figure 5D) and PCoA (Figure 5E) clusters based on the unweighted UniFrac distance showed that the microbiota community was related to the dosage of organic zinc. Briefly, the community between the control group and the S120 group and between the O90 group and the O120 group were similar, respectively. The microbiota community of the O30 group was slightly farther from the control and S120 groups. The O60 group had a farther distance from the control and S120 groups. The O90 and O120 groups together had the furthest distance from the control and S120 groups.

**Figure 6:**
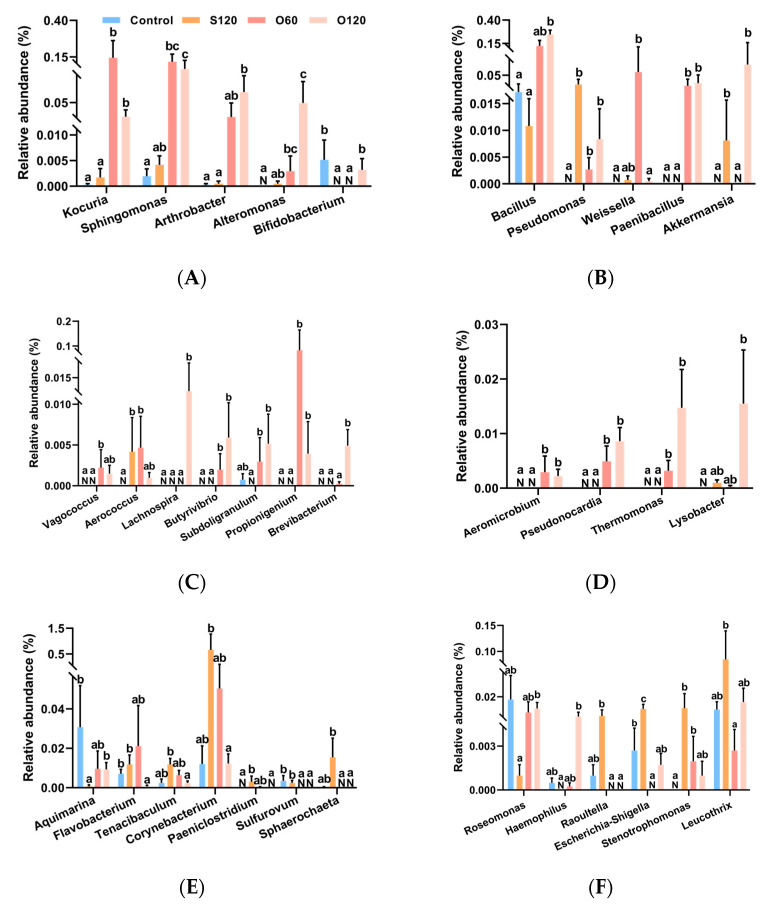

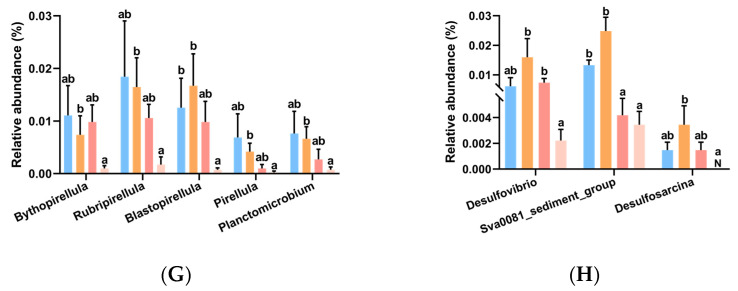
MetaStat analysis of intestinal microbiota communities at genus level of shrimp among control, S120, O60 and O120 groups. (**A**–**D**) potentially beneficial bacteria of shrimp intestine; (**E**–**H**) potentially pathogens of shrimp intestine. ^a,b,c^ Value bars not sharing the same superscript letter are significantly different (*Q <* 0.05). “N” means the abundance of genus is too low to get a value at a certain number or the genus does not exist.

MetaStat analysis (Figure 6 and Table S5) showed that forty-two significantly changed genera were obtained among the control, S120, O60 and O120 groups, of which 21 genera might be beneficial to the host (Figure 6A–D) (e.g., *Aeromicrobium*, *Arthrobacter*, *Butyrivibrio*, *Kocuria* and *Sphingomonas*), and of which 21 genera might be pathogenic to the host (Figure 6E–H) (e.g., *Corynebacterium*, *Escherichia-Shigella*, *Flavobacterium*, *Sva0081_sediment_group* and *Rubripirellula*). Briefly, the genera in Figure 6A,B were potential probiotics for the host. The genera in Figure 6C produce organic acid (e.g., short-chain fatty acid, lactic acid). The genera in Figure 6D produce antibiotics or have antibacterial activity. The genera in Figure 6E–G are potential pathogens for the host or cause diseases. The genera in Figure 6H reduce sulfate into hydrogen sulfide. According to the result, compared with the control diet, the abundance of the majority of potential beneficial genera were significantly increased by the O60 (13 of 21) and O120 (16 of 21) diets (Table S6) (*Q* < 0.05). Compared with the S120 diet, O120 diet significantly increased the abundance of most potential beneficial genera (16 of 21) and decreased the abundance of many potential harmful genera (16 of 21) (Table S6) (*Q* < 0.05).

## 4. Discussion

Some studies in channel catfish [20], pangasius catfish [23], juvenile abalone [24] and white shrimp [27] have shown benefits of organic zinc on growth. However, in the present study, white shrimp fed either organic or inorganic zinc had similar growth performance, concurrent with previous studies in beluga sturgeon [22], rainbow trout [49], Atlantic salmon (*Salmo salar*) [50] and European sea bass (*Dicentrarchus labrax*) [51]. The inconsistent results might be related to different species, developmental stages, diet formulation, culture condition and culture duration.

Investigations on zinc accumulation and transport accumulation have suggested that organic zinc has higher facilitation effects than inorganic zinc. In the present study, zinc concentrations of muscle and carapace were not affected. However, zinc concentrations of hepatopancreas and plasma were significantly higher with dietary zinc. Moreover, organic zinc showed significantly higher levels of zinc concentrations of hepatopancreas and plasma compared with inorganic zinc. This was similar to a previous study on white shrimp, where hepatopancreatic zinc was affected by dietary zinc and phytic acid, while carapace zinc was not [11]. This might also be related to the essential role of zinc in regulating antioxidant defense systems and immune responses, as well as in maintaining nutrient metabolism and transport, since the hepatopancreas and hemolymph are the main sites where these biological processes occur [3]. Additionally, zinc concentration regulation is closely related to two zinc transporter proteins: ZnTs (Slc30a family), which regulate the efflux of zinc from the cytoplasm [52,53], and ZIPs (Slc39a family), which regulate the influx of zinc into the cytoplasm [54]. MT is a cysteine-rich intracellular metal-binding protein that is inducible by Zn^2+^ [55]. By binding and releasing Zn^2+^, MT regulates intracellular Zn^2+^ homeostasis to prevent the cytotoxicity of excess Zn^2+^ [56]. In the present study, organic zinc resulted in greater promotion of ZnT1, ZIP11 and MT expressions in the hepatopancreas than inorganic zinc, which was consistent with the changes in zinc concentrations of the hepatopancreas and plasma, suggesting that organic zinc could increase the intracellular concentration and transport of Zn^2+^ and maintain Zn^2+^ homeostasis. Moreover, zinc homeostasis, in which ZnTs, ZIPs and MT are involved, contributes to a range of physiological functions, including antioxidant capacity and immunity [57,58].

The role of zinc in enhancing antioxidant capacity has been widely summarized [2,4,59,60,61]. Previous studies have also demonstrated improved antioxidant capacity of zinc in beluga sturgeon [22], pangasius catfish [23], triploid trout [25] and white shrimp [29]. Antioxidant reactions are important for cells to prevent damage from free radicals, and the primary enzymatic systems include SOD (Cu/Zn SOD is the most prominent), CAT and Gpx [61]. SOD converts oxygen radicals into H_2_O_2_, which in turn is catalyzed by CAT or Gpx into H_2_O and O_2_ [61,62]. SOD is the most important and powerful oxidative defense enzyme and requires zinc as a cofactor for its activity [61]. T-AOC is defined as the capacity of inhibition of lipid oxidative degradation, which is important for inhibiting free radical production or maintaining antioxidant activity [63]. Moreover, it has been reported that zinc can decrease MDA content in cells [59]. On the other hand, MT releases Zn^2+^, which in turn activates metal-responsive transcription factor-1 intracellularly and subsequently upregulates the expressions of a variety of antioxidant genes, including Gpx, GST, G6PDH and MT [60]. This is consistent with the present study, in which dietary zinc enhanced the antioxidant capacity of shrimp plasma and hepatopancreas, as evidenced by increased activities of T-SOD, Cu/Zn SOD, CAT and T-AOC, upregulated CAT, Gpx, GST and G6PDH expressions, and decreased MDA content. However, organic zinc, especially in the O60, O90 and O120 groups, had better efficacy. The present study revealed that organic zinc was better than inorganic zinc in enhancing antioxidant capacity, which might be related to the improved Zn^2+^ homeostasis.

Zinc is well known for its important role in immune response [64]. ACP and AKP are typically used to assess the immune status of invertebrates [65]. PO, LZM and Hemo are crucial innate immune defense molecules for shrimp and take part in various immune responses, including against pathogens [66,67,68,69]. Some studies have reported the advantages of organic zinc in improving immunity in channel catfish [21], beluga sturgeon [22], pangasius catfish [23] and white shrimp [27,29]. In the present study, increased activities (ACP, AKP, PO and LZM) and expressions (ACP, ProPO, LZM and Hemo) demonstrated beneficial changes in the immune system with organic zinc, which were also consistent with lower mortality during the *V*. *parahaemolyticus* challenge. The mechanism might be that a strengthened intracellular zinc homeostasis status allows Zn^2+^ to regulate distinct signaling pathways in immune systems more positively to influence cellular activities such as immune cells [70].

Zinc can also stimulate glucose transport [71] and glycolysis [72]. Glucose is absorbed from the intestinal lumen into the hemolymph via glucose transporter (GluT) and transported to the hepatopancreas via the open circulatory system [73]. In the hepatopancreas, Glu is converted into pyruvate via glycolysis [74], and then pyruvate enters the TAC via the PDH complex for further energy production [75]. GCK and PFK are rate-limiting and key regulatory enzymes of glycolysis reactions [74,76]. The increased GluT1 expression, Glu content, GCK and PFK activities of the present results revealed that organic zinc supported energy production from glucose to a greater degree than inorganic zinc. Changes in glucose transport and glycolysis might be related to the fact that zinc is involved in the secretion of insulin and glucagon [77]. The decreased pyruvate content might be due to the increased PDH activity that facilitates the entry of pyruvate into the tricarboxylic acid cycle for further energy production [75]. Similarly, previous studies in rats and mice have also reported the role of zinc in stimulating glycolysis [30,31,78]. However, the specific mechanism by which zinc regulates glucose metabolism in white shrimp needs further study.

The intestinal microbiota community of shrimp is tightly associated with its health [79]. Few studies have reported the effects of zinc on intestinal microbiota in shrimp. The present study found that Firmicutes, Proteobacteria and Bacteroidota were the predominant phyla, and *Candidatus_Bacilloplasma*, *Vibrio* and *Spongiimonas* were the predominant genera, which is consistent with previous results in shrimp [80,81]. Moreover, the cluster results showed that the microbiota community was migrated by the dosage of organic zinc, especially in the O60, O90 and O120 groups, since the top 10 bacteria were similar among all groups and alpha diversity did not differ greatly. The migration might be caused by changes in the abundance of non-predominant bacteria. Forty-two significantly different genera were observed by MetaStat analysis. When compared with the control group, only 7 genera were observed in the S120 group, while about 20 genera were observed in the O60 or O120 groups. When compared with inorganic zinc (S120), organic zinc (O60 and O120)-supplemented shrimp had a significantly higher abundance of potentially beneficial bacteria yet lower abundance of potentially pathogenic bacteria. Among these genera, some probiotics have been used in aquaculture such as *Kocuria*, *Sphingomonas*, *Arthrobacter*, *Alteromonas*, *Bifidobacterium*, *Bacillus*, *Pseudomonas*, *Weissella* and *Paenibacillus* [82,83,84]. Some probiotics include lactic acid producers (*Akkermansia*, *Vagococcus*, *Aerococcus*) [85,86] and short-chain fatty acid producers (*Lachnospira*, *Butyrivibrio*, *Subdoligranulum*, *Propionigenium*, *Brevibacterium*) [87,88,89,90,91]. The presence of these probiotics in the present study may be beneficial to the nutrient availability, antioxidant and immune response of white shrimp [91]. Increased *Aeromicrobium*, *Pseudonocardia*, *Thermomonas* and *Lysobacter*, which could produce secondary metabolites and have antibacterial properties, may protect shrimp from pathogens [92,93,94,95]. Moreover, the bacteria *Aquimarina*, *Tenacibaculum*, *Flavobacterium*, *Paeniclostridium*, *Escherichia-Shigella*, *Sulfurovum* and *Leucothrix* are opportunistic or true pathogens that cause diseases of aquatic animals and egg mortality [96,97,98,99]. Some species in *Corynebacterium*, *Sphaerochaeta*, *Roseomonas*, *Haemophilus*, *Raoultella* and *Stenotrophomonas* are also potentially pathogenic agents of the host [100,101,102,103,104]. *Bythopirellula*, *Rubripirellula*, *Blastopirellula*, *Pirellula* and *Planctomicrobium* are related to host diseases and resistant to a variety of antibiotics [105]. *Desulfovibrio*, *Sva0081_sediment_group* and *Desulfosarcina* are sulfate-reducing bacteria that reduce sulfate into hydrogen sulfide [106,107,108]. The high concentration of hydrogen sulfide is toxic to intestinal microbiota and lowers intestinal pH [108]. This also prompts us to be concerned about the potential risks in the application of sulfate mineral elements. Overall, the present study suggested that organic zinc-fed shrimp had a higher abundance of potentially beneficial genera and lower abundance of potential pathogens in intestinal samples of white shrimp. Taken in combination with other findings of this study, it is possible that the shrimp might have evolved a mechanism to regulate intestinal microbiota balance by utilizing zinc homeostasis [109]. On the one hand, dietary zinc is used by the host to inhibit the proliferation of pathogens and regulate interspecies competition of intestinal microbiota [110]. On the other hand, dietary zinc can enhance intestinal mucosal barrier function, which is beneficial for the colonization of probiotics and defense against pathogens [111,112]. The organic zinc supplementation benefited zinc homeostasis of white shrimp, which was crucial for enhancing the healthy balance of host and intestinal microbes [113].

## 5. Conclusions

The present results showed that both inorganic zinc and organic zinc could benefit the antioxidant capacity and immune response of shrimp. However, compared with inorganic zinc, organic zinc could further support zinc accumulation and transport, enhance antioxidant capacity and immune response, stimulate glucose transport and glycolysis and strengthen the resistance to disease of shrimp. Moreover, organic zinc resulted in beneficial shifts in the intestinal microbiota community, with a higher abundance of various potentially beneficial bacteria and a lower abundance of several potentially harmful bacteria. Overall, shrimp fed 60 mg·kg^−1^ zinc from Zn-proteinate achieved better effects than those fed 120 mg·kg^−1^ zinc from ZnSO_4_·7H_2_O. The mechanism of organic zinc in these effects might be related to the improving zinc homeostasis of shrimp, which indicated that organic zinc has higher bioavailability than inorganic zinc in shrimp feeds.

## Figures and Tables

**Table 1:** Formulation and proximate compositions of experimental diets.

Ingredients (%)	Diets					
	Control	S120	O30	O60	O90	O120
Fish meal ^1^	15.00	15.00	15.00	15.00	15.00	15.00
Shrimp shell meal ^1^	5.00	5.00	5.00	5.00	5.00	5.00
Brewer yeast ^1^	5.00	5.00	5.00	5.00	5.00	5.00
Soybean meal ^1^	30.00	30.00	30.00	30.00	30.00	30.00
Cottonseed protein ^1^	5.00	5.00	5.00	5.00	5.00	5.00
Peanut meal ^1^	10.00	10.00	10.00	10.00	10.00	10.00
Wheat flour ^1^	22.00	22.00	22.00	22.00	22.00	22.00
Fish oil ^1^	1.00	1.00	1.00	1.00	1.00	1.00
Soybean oil ^1^	1.00	1.00	1.00	1.00	1.00	1.00
Phospholipid ^1^	1.00	1.00	1.00	1.00	1.00	1.00
Monocalcium phosphate ^1^	1.00	1.00	1.00	1.00	1.00	1.00
Choline chloride ^2^	0.20	0.20	0.20	0.20	0.20	0.20
Vitamin mix ^3^	1.00	1.00	1.00	1.00	1.00	1.00
Mineral mix (Zn Free) ^3^	1.00	1.00	1.00	1.00	1.00	1.00
Lysine hydrochloride ^1^	0.10	0.10	0.10	0.10	0.10	0.10
Methionine ^2^	0.10	0.10	0.10	0.10	0.10	0.10
Threonine ^2^	0.05	0.05	0.05	0.05	0.05	0.05
Vitamin C-35 phosphate ^1^	0.20	0.20	0.20	0.20	0.20	0.20
ZnSO_4_·7H_2_O (22.74%) ^2^	-	0.0528	-	-	-	-
Bioplex Zn^®^ (15%) ^4^	-	-	0.02	0.04	0.06	0.08
Astaxanthin (10%) ^1^	0.10	0.10	0.10	0.10	0.10	0.10
Y_2_O_3_ ^2^	0.01	0.01	0.01	0.01	0.01	0.01
Carrier ^1^	1.24	1.1872	1.22	1.20	1.18	1.16
Analyzed Nutrient Compositions (% Dry Matter)						
Crude protein	45.70	45.76	45.96	46.22	45.89	46.46
Crude lipid	4.32	4.48	4.41	4.41	4.72	4.67
Ash	8.92	9.16	9.36	9.13	9.23	9.25
Zinc Analysis (mg·kg^−^^1^)						
Zn (formulated value)	0	120	30	60	90	120
Zn (analyzed value)	53	133	86	106	138	171

^1^ Fish meal, shrimp shell meal, brewer yeast, etc., were purchased from Qingdao Great-seven Nutr-tech Co., Ltd., Qingdao, China. ^2^ Choline chloride, amino acid, ZnSO_4_·7H_2_O and Y_2_O_3_ were purchased from Shanghai Macklin Biochemical Co., Ltd., Shanghai, China. ^3^ Vitamin premix and mineral premix were purchased from Qingdao Master Biotech Co., Ltd., Qingdao, China. Vitamin premix contains (kg^−1^): vitamin A acetate, 714,000 IU; vitamin D3, 266,000 IU; DL-α-tocopherol acetate, 8.6 g; menadione, 1.0 g; thiamine mononitrate, 1.0 g; riboflavin, 1.4 g; pyridoxine hydrochloride, 1.2 g; cyanocobalamin, 0.004 g; D-calcium pantothenate, 4.0 g; nicotinamide, 6.8 g; folic acid, 0.28 g; D-biotin, 0.012 g; inositol, 7.6 g; L-ascorbic acid-2-phosphate, 16.6 g; mineral premix contains (kg^−1^): Mg, 12.5 g; Fe, 4.0 g; Mn, 2.0 g; Cu, 1.25 g; Co, 0.05 g; Se, 0.015 g; I, 0.05 g. ^4^ Bioplex Zn^®^ was provided by Beijing Alltech Biological Products (China) Co., Ltd., Beijing, China.

**Table 2:** Effects of organic and inorganic zinc on growth performance of *Litopenaeus vannamei* Boone, 1931 *.

Diets	Control	S120	O30	O60	O90	O120
IBW (g)	2.34 ± 0.02	2.38 ± 0.02	2.38 ± 0.03	2.36 ± 0.02	2.35 ± 0.02	2.38 ± 0.01
FBW (g)	10.02 ± 0.32 ^a^	11.25 ± 0.43 ^ab^	10.56 ± 0.18 ^ab^	12.41 ± 0.83 ^b^	10.50 ± 0.32 ^ab^	10.73 ± 0.39 ^ab^
WGR (%)	328.3 ± 13.1 ^a^	374.0 ± 20.1 ^ab^	343.9 ± 12.0 ^ab^	427.2 ± 36.2 ^b^	348.0 ± 16.2 ^ab^	351.6 ± 16.0 ^ab^
SGR (%·day^−1^)	2.59 ± 0.06 ^a^	2.77 ± 0.08 ^ab^	2.66 ± 0.05 ^ab^	2.96 ± 0.12 ^b^	2.67 ± 0.07 ^ab^	2.69 ± 0.06 ^ab^
FI (%·day^−1^)	1.56 ± 0.04	1.43 ± 0.05	1.47 ± 0.04	1.33 ± 0.07	1.44 ± 0.09	1.54 ± 0.08
FE	0.357 ± 0.014	0.409 ± 0.022	0.384 ± 0.013	0.463 ± 0.040	0.399 ± 0.029	0.372 ± 0.024
CF (100 g·cm^−3^)	0.652 ± 0.004	0.658 ± 0.010	0.650 ± 0.007	0.650 ± 0.003	0.648 ± 0.007	0.637 ± 0.011
Total hemocyte (×10^6^)	27.14 ± 1.35	24.18 ± 1.90	26.25 ± 2.02	28.64 ± 2.11	24.42 ± 2.26	22.51 ± 2.42

* Values represent are means ± S.E. of 4 replicate tanks. IBW, initial body weight, FBW, final body weight, WGR, weight gain rate, SGR, specific growth rate, FI, feed intake, FE, feed efficiency, CF, condition factor. ^a,b^ Different superscript letters within a row denote significant differences as evaluated by Tukey’s test (*p <* 0.05).

**Table 3:** Richness and diversity indices of intestinal microbiota of *L. vannamei*. *.

Diets	Control	S120	O30	O60	O90	O120
OTUs	281 ± 25	346 ± 33	386 ± 47	336 ± 17	253 ± 39	281 ± 21
Chao1	310.7 ± 22.9 ^ab^	385.4 ± 35.7 ^ab^	422.0 ± 48.2 ^b^	379.1 ± 16.9 ^ab^	278.1 ± 40.0 ^a^	303.1 ± 21.4 ^ab^
ACE	332.8 ± 21.3 ^ab^	404.8 ± 36.7 ^ab^	445.7 ± 47.5 ^b^	403.5 ± 15.7 ^ab^	292.6 ± 41.3 ^a^	319.8 ± 21.0 ^ab^
Shannon	2.66 ± 0.18	3.05 ± 0.20	3.35 ± 0.30	2.99 ± 0.10	2.70 ± 0.16	3.24 ± 0.10
Simpson	0.693 ± 0.045 ^a^	0.757 ± 0.026 ^ab^	0.801 ± 0.023 ^ab^	0.768 ± 0.016 ^ab^	0.695 ± 0.028 ^a^	0.812 ± 0.012 ^b^
PD whole tree	18.61 ± 1.00	22.64 ± 1.45	24.10 ± 1.89	22.60 ± 0.81	19.07 ± 2.33	20.67 ± 1.00

* Values represent are means ± S.E. of 4 replicate tanks. ^a,b^ Different superscript letters within a row denote significant differences as evaluated by Tukey’s test (*p <* 0.05).

## Data Availability

The data of 16S rRNA sequence presented in the study are deposited in https://www.ncbi.nlm.nih.gov/sra (accessed on 3 May 2022), under Accession Number PRJNA834576.

## Supplementary Materials

The following supporting information can be downloaded at: www.mdpi.com/article/10.3390/antiox11081492/s1, Figure S1. Rank abundance (A), rarefaction curves (B) and species accumulation boxplot (C) for all the intestinal microbiota samples; Table S1. Primers used in qPCR and amplification information; Table S2. Descriptive statistics of 3 candidate housekeeping genes (HKG) based on their cycle quantification (Cq) values; Table S3. Stability analysis of 3 candidate HKG based on their Cq values; Table S4. AMOVA analysis based on unweighted UniFrac distance of microbial community structure of *Litopenaeus vannamei* Boone, 1931; Table S5. The genus level classification information of MetaStat analysis showed in Figure 6; Table S6. The genera abundance changes of *L. vannamei* based on MetaStat analysis.
