# Supplementary material for: The Assessment of Dietary Organic Zinc on Zinc Homeostasis, Antioxidant Capacity, Immune Response, Glycolysis and Intestinal Microbiota in White Shrimp (Litopenaeus vannamei Boone, 1931)
Source: Antioxidants (Basel). 2022 Jul 29;11(8):1492. doi: 10.3390/antiox11081492 (PMC9405169; doi:10.3390/antiox11081492)
Supplement: Supplementary file 1 [file antioxidants-11-01492-s001.zip › antioxidants-1792216-supplementary.pdf]

antioxidants

Supplementary Materials: The Assessment of Dietary Organic Zinc on Zinc Homeostasis, Antioxidant Capacity, Immune Response, Glycolysis and Intestinal Microbiota in White Shrimp (*Litopenaeus vannamei* Boone, 1931)

Jinzhu Yang [1], Tiantian Wang [1], Gang Lin [2], Mingzhu Li [3], Yanjiao Zhang [1,*] and Kangsen Mai [1]

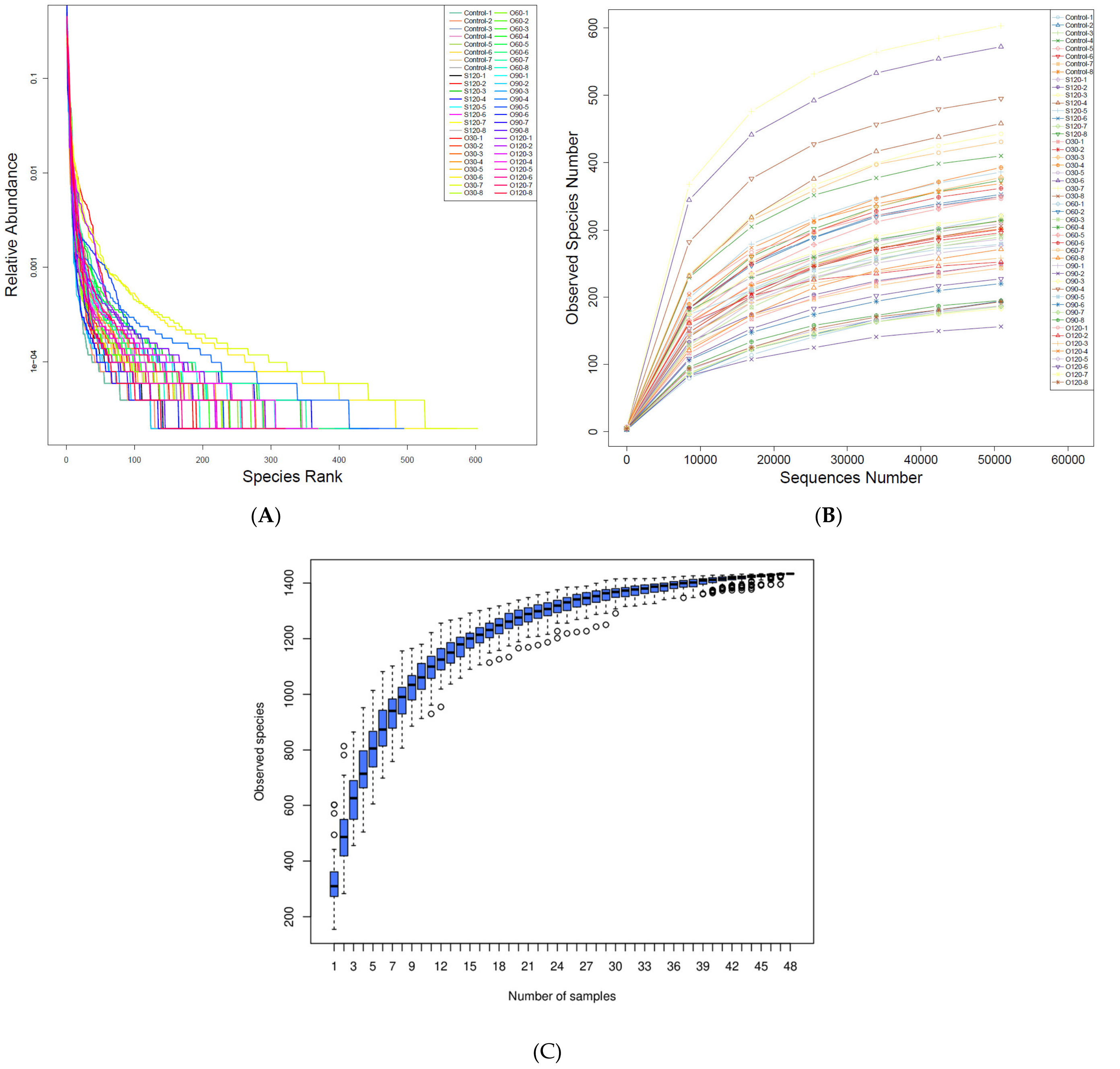

Figure S1. Rank abundance (**A**), Rarefaction curves (**B**) and Species accumulation boxplot (**C**) for all the intestinal microbiota samples.

Table S1. Primers used in qPCR and amplification information.

Genes	Primer sequence (5'-3')		Tm (°C)	Product length	Amplification efficiency	GenBank Accession no.
	Forward	Reverse				
β-Actin	CGAGAGGAAGCAGCACGTA	GACGATGGAGGGGAACACAG	60	164	1.01	AF300705.2
EF1α	TCGCCTTCTATCATCCCCATC	GATGTTGTCCAGGGCATCGAAC	60	147	1.08	JF288785.1
18S rRNA	CTGCGAAAGCATCTGCCAAG	GGATCGCTAGTCAGCATCGT	60	113	0.94	AF186250.1
CAT	TAAGGGAGCAGGTGCCTTTG	ATCCCTGGCAGTGTCAGTTG	60	157	1.00	AY518322.1
G6PDH	ACATCGCCTCGGTCTTCATC	GAACTCGTCGAAGTAGCCGC	60	74	1.06	MK814533.1
Gpx	GGCACCAGGAGAACACTACC	TCGAAGTTGTTCCCAGGACG	60	73	1.03	AY973252.2
GST	GAATGCTGCCCGTTTTAGGC	CCGAGTTTGAACTTGACGGC	60	163	1.06	AY573381.2
SOD	ACGTAAGCGCAATGAATGCC	GAAGCCATGTTGGGTCCAGA	60	93	1.09	AB108065.1
ACP	AAGCCTGAAGTTCGTGCTGA	TGACTCTGGTGCAGTCATCG	60	97	1.03	KR676449.1
AKP	GCGAGACGACAACGGATTC	CAGCGGTGACGATGATAAGAG	60	170	1.11	KR534873.1
Hemo	AGACTGGGCATCCTTTGTCG	TCATAGAGGGGAGGGAGCAC	60	135	1.06	MK896907.1
LZM	GGTGCGCCGAGACTATCC	TTGCTGTTGTAAGCCACCCA	60	85	1.07	AY170126.2
ProPO	GAACTCCATTCCGTCCGTCTG	GGCTTCGCTCTGGTTAGGAT	60	123	1.05	AY723296.1
Glut1	CTACTCGCATGCTTCTGGGT	GTCGCGCTTCTCCTCTAGGT	60	150	1.00	KM201335.1
MT	CCCATCCAAGGAGGAGTGTG	AGCAGCAGAAGACAGTCGAG	60	88	1.03	JN707684.1
ZnT1	TTCCAGGAGCCTCAAAGCAG	GTAGAGCGCTATGGTTCCCC	60	148	0.97	QCYY01003491.1
ZIP11	CGGAAGGCATGGCAGTTAGT	GCTCCCAGAACACCTGCTAC	60	115	1.05	XM_027361504.1

Abbreviations: EF1α, elongation factor 1-alpha; 18S rRNA, 18S ribosomal RNA; CAT, catalase; G6PDH, glucose-6-phosphate dehydrogenase; Gpx, glutathione peroxidase; GST, glutathione S-transferase; SOD, superoxide dismutase; ACP, acid phosphatase; AKP, alkaline phosphatase; Hemo, hemocyanin; LZM: lysozyme; ProPO, pro-phenoloxidase; Glut1, glucose transporter 1; MT, metallothionein; ZnT1, zinc transporter 1; ZIP11, Zrt-/Irt-like protein 11.

Table S2 description was gain from Excel based tool BestKeeper [1]. Briefly, all cycle quantification (Cq) values of 3 candidate housekeeping genes were inputted in the BestKeeper on Excel table. The main calculation results were obtained as shown in Table S2. Any gene with the std dev [± Cq] value lower than 1.00 was considered stably expressed. Furthermore, based on the std dev [± Cq] value and CV [% Cq] value, any gene with the lowest value was considered the most stably expressed, which meant that β-actin was the best housekeeping gene in the present experiment.

Table S3 description was gain from Excel add-in "NormFinder.xla" [2]. Briefly, all cycle quantification (Cq) values of 3 candidate housekeeping genes were inputted in Excel table. After edited the gene name and group catalog, "NormFinder" from Excel add-ins was selected. Then all relative data were selected using the dialog box. Finally, the main output data were shown in Table S3. By comparing the stability values of 3 candidate housekeeping genes, any gene with the lowest value was considered the most stably expressed, which meant that β-actin was the best housekeeping gene in the present experiment.

Finally, β-actin was assessed as the best housekeeping gene in the present experiment.

Table S2. Descriptive statistics of 3 candidate housekeeping genes (HKG) based on their cycle quantification (Cq) values.

Factor	β-Actin	EF1α	18S rRNA
GM [Cq]	17.01	23.15	9.57
AM [Cq]	17.02	23.19	9.58
Min [Cq]	16.52	20.69	8.40
Max [CP]	17.50	25.67	10.26
std dev [± Cq]	0.25	1.15	0.38
CV [% Cq]	1.46	4.97	3.95
Min [x-fold]	-1.41	-6.09	-2.17
Max [x-fold]	1.40	6.34	1.58
std dev [± x-fold]	1.19	2.24	1.30

Abbreviations: GM [Cq]: the geometric mean of Cq; AM [Cq]: the arithmetic mean of Cq; Min [Cq] and Max [Cq]: the extreme values of Cq; std dev [± Cq]: the standard deviation of the Cq; CV [% Cq]: the coefficient of variance expressed as a percentage on the Cq level; Min [x-fold] and Max [x-fold]: the extreme values of expression levels expressed as an absolute x-fold over- or under-regulation coefficient; std dev [± x-fold]: standard deviation of the absolute regulation coefficient.

Table S3. Stability analysis of 3 candidate HKG based on their Cq values.

Gene name	β-actin	EF1α	18S rRNA
Stability value	0.312	0.649	0.445
Best HKG		β-actin	

Reference

1. Pfaffl, M.W.; Tichopad, A.; Prgomet, C.; Neuvians, T.P. Determination of Stable Housekeeping Genes, Differentially Regulated Target Genes and Sample Integrity: BestKeeper--Excel-Based Tool Using Pair-Wise Correlations. *Biotechnol. Lett.* **2004**, *26*, 509–515.
2. Andersen, C.L.; Jensen, J.L.; Ørntoft, T.F. Normalization of Real-Time Quantitative Reverse Transcription-PCR Data: A Model-Based Variance Estimation Approach to Identify Genes Suited for Normalization, Applied to Bladder and Colon Cancer Data Sets. *Cancer Res.* **2004**, *64*, 5245–5250.

Table S4. Amova analysis based on Unweighted Unifrac distance of microbial community structure of *Litopenaeus vannamei* Boone, 1931.

Groups	*P*
Control-S120	<0.001*
Control-O30	<0.001*
Control-O60	<0.001*
Control-O90	<0.001*
Control-O120	<0.001*
S120-O30	<0.001*
S120-O60	<0.001*
S120-O90	<0.001*
S120-O120	<0.001*
O30-O60	<0.001*
O30-O90	<0.001*
O30-O120	<0.001*
O60-O90	<0.001*
O60-O120	<0.001*
O90-O120	0.048*

P value smaller than 0.05 indicated the difference between two groups was significant.

Table S5. The genus level classification information of MetaStat analysis showed in Figure 6.

Phylum	Class	Order	Family	Genus	Location
Actinobacteriota	Actinobacteria	Micrococcales	Micrococcaceae	*Kocuria*	Figure 6A
Actinobacteriota	Actinobacteria	Micrococcales	Micrococcaceae	*Arthrobacter*	Figure 6A
Actinobacteriota	Actinobacteria	Bifidobacteriales	Bifidobacteriaceae	*Bifidobacterium*	Figure 6A
Proteobacteria	Alphaproteobacteria	Sphingomonadales	Sphingomonadaceae	*Sphingomonas*	Figure 6A
Proteobacteria	Gammaproteobacteria	Alteromonadales	Alteromonadaceae	*Alteromonas*	Figure 6A
Firmicutes	Bacilli	Bacillales	Bacillaceae	*Bacillus*	Figure 6B
Firmicutes	Bacilli	Lactobacillales	Leuconostocaceae	*Weissella*	Figure 6B
Firmicutes	Bacilli	Paenibacillales	Paenibacillaceae	*Paenibacillus*	Figure 6B
Proteobacteria	Gammaproteobacteria	Pseudomonadales	Pseudomonadaceae	*Pseudomonas*	Figure 6B
Verrucomicrobiota	Verrucomicrobiae	Verrucomicrobiales	Akkermansiaceae	*Akkermansia*	Figure 6B
Actinobacteriota	Actinobacteria	Micrococcales	Brevibacteriaceae	*Brevibacterium*	Figure 6C
Firmicutes	Bacilli	Lactobacillales	Vagococcaceae	*Vagococcus*	Figure 6C
Firmicutes	Bacilli	Lactobacillales	Aerococcaceae	*Aerococcus*	Figure 6C
Firmicutes	Clostridia	Lachnospirales	Lachnospiraceae	*Lachnospira*	Figure 6C
Firmicutes	Clostridia	Lachnospirales	Lachnospiraceae	*Butyrivibrio*	Figure 6C
Firmicutes	Clostridia	Oscillospirales	Ruminococcaceae	*Subdoligranulum*	Figure 6C
Fusobacteriota	Fusobacteriia	Fusobacteriales	Fusobacteriaceae	*Propionigenium*	Figure 6C
Actinobacteriota	Actinobacteria	Propionibacteriales	Nocardioidaceae	*Aeromicrobium*	Figure 6D
Actinobacteriota	Actinobacteria	Pseudonocardiales	Pseudonocardiaceae	*Pseudonocardia*	Figure 6D
Proteobacteria	Gammaproteobacteria	Xanthomonadales	Xanthomonadaceae	*Thermomonas*	Figure 6D
Proteobacteria	Gammaproteobacteria	Xanthomonadales	Xanthomonadaceae	*Lysobacter*	Figure 6D
Actinobacteriota	Actinobacteria	Corynebacteriales	Corynebacteriaceae	*Corynebacterium*	Figure 6E
Bacteroidota	Bacteroidia	Flavobacteriales	Flavobacteriaceae	*Aquimarina*	Figure 6E

Bacteroidota	Bacteroidia	Flavobacteriales	Flavobacteriaceae	*Flavobacterium*	Figure 6E
Bacteroidota	Bacteroidia	Flavobacteriales	Flavobacteriaceae	*Tenacibaculum*	Figure 6E
Campilobacterota	Campylobacteria	Campylobacterales	Sulfurovaceae	*Sulfurovum*	Figure 6E
Firmicutes	Clostridia	Peptostreptococcales-Tissierellales	Peptostreptococcaceae	*Paeniclostridium*	Figure 6E
Spirochaetota	Spirochaetia	Spirochaetales	Spirochaetaceae	*Sphaerochaeta*	Figure 6E
Proteobacteria	Alphaproteobacteria	Acetobacterales	Acetobacteraceae	*Roseomonas*	Figure 6F
Proteobacteria	Gammaproteobacteria	Pasteurellales	Pasteurellaceae	*Haemophilus*	Figure 6F
Proteobacteria	Gammaproteobacteria	Enterobacterales	Enterobacteriaceae	*Raoultella*	Figure 6F
Proteobacteria	Gammaproteobacteria	Enterobacterales	Enterobacteriaceae	*Escherichia-Shigella*	Figure 6F
Proteobacteria	Gammaproteobacteria	Xanthomonadales	Xanthomonadaceae	*Stenotrophomonas*	Figure 6F
Proteobacteria	Gammaproteobacteria	Thiotrichales	Thiotrichaceae	*Leucothrix*	Figure 6F
Planctomycetes	unidentified_Planctomycetes	Pirellulales	Pirellulaceae	*Bythopirellula*	Figure 6G
Planctomycetes	unidentified_Planctomycetes	Pirellulales	Pirellulaceae	*Rubripirellula*	Figure 6G
Planctomycetes	unidentified_Planctomycetes	Pirellulales	Pirellulaceae	*Blastopirellula*	Figure 6G
Planctomycetes	unidentified_Planctomycetes	Pirellulales	Pirellulaceae	*Pirellula*	Figure 6G
Planctomycetes	unidentified_Planctomycetes	Planctomycetales	Rubinisphaeraceae	*Planctomicrobium*	Figure 6G
Desulfobacterota	Desulfovibrionia	Desulfovibrionales	Desulfovibrionaceae	*Desulfovibrio*	Figure 6H
Desulfobacterota	Desulfobacteria	Desulfobacterales	Desulfosarcinaceae	*Sva0081_sediment_group*	Figure 6H
Desulfobacterota	Desulfobacteria	Desulfobacterales	Desulfosarcinaceae	*Desulfosarcina*	Figure 6H

Table S6. The genera abundance changes of *L. vannamei* based on MetaStat analysis

↑ or ↓ means the abundance was significantly increased or decreased when compared with Control, S120 and O60 groups; number in the cells indicates the number of significantly changed genera.

	**vs Control**			**vs S120**		**vs O60**
A-D	**S120**	**O60**	**O120**	**O60**	**O120**	**O120**
↑	3	13	16	9	16	4
↓	0	1	0	1	0	1
E-H	S120	O60	O120	O60	O120	O120
↑	3	1	0	0	3	0
↓	1	3	4	6	16	1
